# Development of a Hands-On and Virtual Simulation Training Module To Teach Microtomy

**DOI:** 10.7759/cureus.25720

**Published:** 2022-06-07

**Authors:** Samira Wahab, Dania Buttu, Donna Smeeton, Adam Dubrowski

**Affiliations:** 1 Health Sciences, Ontario Tech University, Oshawa, CAN

**Keywords:** simulation based training, delphi method, skills and simulation training, simulation in medical education, microtomy

## Abstract

Background: Microtomy is a risky procedure that medical laboratory technologists (MLTs) use to cut tissue samples for microscopic examination. Due to the safety concerns and the potential to destroy tissue samples, it is critical for learners to perform the procedure correctly. To allow for safe and controlled learning, this procedure should be acquired in a safe and controlled simulated setting before being attempted on human tissues. The overarching purpose of this work is the development of a virtual training module for undergraduate students to learn from. However, because of the heterogeneity in the steps required to successfully complete the procedure from the MLTs as well as in the literature, the aim of this study was to reach a consensus from a panel of experts about identifying the steps of the procedure using the think-aloud and modified-Delphi methods.

Methods: First, we conducted a think-aloud protocol with a single MLT expert trained in microtomy to generate the list of steps of the microtomy procedure objectively. In order to remove any idiosyncratic steps, next, we asked eight experts that were trained in histology to rate the criticalness of each step using a (1-5) Likert scale and provide evaluative feedback.

Results: The think-aloud protocol generated 10 steps for the microtomy procedure. During the subsequent two rounds of the Delphi exercise, the experts agreed to modify one step of the 10 steps.

Conclusions: Through this work, the 10 steps of the microtomy procedure have been validated by experts in the field. Following that, a virtual simulation training module was built to instruct learners on the microtomy procedure. The virtual simulation training module may be used for further research in microtomy.

## Introduction

Microtomy is a process that medical laboratory technologists (MLTs) use to cut tissue samples for further examination, making this a critical process for any sample slide to be examined under a microscope. A "microtome" is a specialized cutting tool used to cut extremely thin slices of tissue samples. When conducting the microtomy procedure, it is vital to understand all the equipment parts and the safety features needed to perform this procedure accurately and safely to minimize damage or distortion to the tissue sample [1]. A series of steps must be completed in sequential order and learners must become aware of the cautious safety features when handling the microtome instrument.

Ensuring that an MLT correctly prepares a sample is critical because if not done correctly, the entire tissue sample is tarnished and must be re-prepared because the tissue specimen can be cut and get damaged, resulting in a poor sample. Using simulation-based education, our overarching aim is to build a virtual simulation training module where the students can practice the procedure multiple times in a safe and controlled environment. However, currently, the literature does not validate the content of the microtomy procedure or provide consistency in the required steps of the microtomy procedure. The inconsistency of the microtomy procedure demonstrated in the literature [1,2] indicates that MLTs are being trained differently, which could potentially result in some MLTs being unaware of crucial steps of the procedure [2].

This study is part one of two in which the objective is to develop a virtual simulation training module to teach the microtomy procedure. To do so, we need to validate the sequential steps to execute the microtomy procedure safely and efficiently, which is the first and current objective of part one of the study. Doing this will contribute to the validation of the steps needed to create the virtual simulation module.

## Materials and methods

This study employs a think-aloud method to decompose the microtomy procedure into subtasks. The think-aloud method asks people to think out loud while performing a task, followed by analyzing the verbal protocols [3]. These protocols are used to decompose complex tasks or procedures into steps known as actions. However, verbal protocols are another term often used as a synonym for thinking aloud. Verbal protocols can be concurrent (thinking aloud) or retrospective, referring to short reports after the completion of a task. They can be subject to individual biases and habits. To ensure that the verbal protocols (i.e., actions) are representative of an MLT standard, we employed a Delphi methodology to reach consensus among experts about the criticality of the protocols (i.e., actions needed to perform the procedure) and their sequence. The Delphi method is recommended for use in the healthcare setting as a reliable means of determining consensus for a defined clinical problem [4]. It begins with experts completing a survey in round one where they can suggest revisions. The changes would be made, and the survey with any revisions would be sent to the same experts in round two to achieve consensus. If consensus cannot be reached in round two, subsequent rounds would occur until consensus is reached [5]. A modified version of the Delphi was prepared prior to it being brought to the other experts. The purpose of this was that we had access to a local MLT expert who could prepare the initial concept document.

Participants 

Expert participants consisted of MLTs from the faculty of Medical Laboratory Sciences at Ontario Tech University (OTU; two participants) and MLTs from the Canadian Society for Medical Laboratory Sciences (CSMLS; six participants) who had previous and/or current experience with performing the microtomy procedure.

Procedure 

For this study, a single teaching faculty expert from OTU participated in the think-aloud protocol. The expert was asked to perform a microtomy while simultaneously being video recorded and talking through the procedure while providing reasoning and thinking. This is also known as knowledge elicitation [3]. These recordings were used to decompose the entire microtomy procedure into subtasks. 

**Figure 1:**
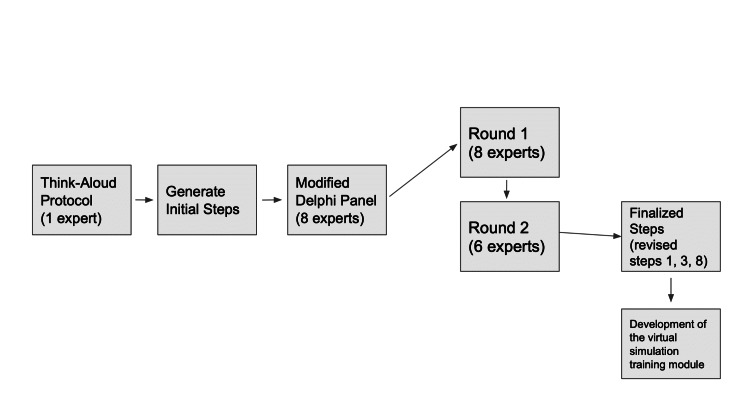
Flow diagram of methodology to develop the virtual simulation training module

Next, a modified Delphi method was used [5]. Eight participants were asked to complete a survey that asked them to rate each of the initial subtasks (i.e., steps of the procedure) as critical (5/5) or not critical (1/5) [5]. Steps with an average of 3.75/5 or above on the scale were considered critical, and therefore they were retained in the list. Those that fell below 3.74/5 were reviewed for variability in the responses. High variability in ratings (criterion 1-point standard deviation (SD)) indicated that the expert may be interpreting the step in various ways [5]. Therefore, more clarity needs to be added to the wording about the step. In this case, written feedback about ways to improve the interpretation of these steps was considered and reviewed. Steps that fell below the 3.74/5 threshold and showed low variability were considered not critical and therefore removed. Based on this algorithm, a new list of steps was generated and submitted to the participants for round two of the modified Delphi method. Typically, using this methodology, consensus is reached within two to three rounds [5]. Figure 1 demonstrates the methodology in a flow diagram to visualize the steps before the virtual simulation training module is developed.

## Results

During the think-aloud protocol, a single expert generated 10 steps (Table 1). These steps served as an initial survey for the subsequent modified Delphi method.

**Table 1:** List of the 10 steps generated from the Think-Aloud method

Step	Description
1.	Place the tissue on a cold surface prior to cutting (cold wax makes the tissue easier to cut).
2.	Fill the water bath and warm to approximately 46 °C.
3.	Ensure the microtome is locked and adhere to microtome safety. Place the blade in the blade holder and cover with the knife guard.
4.	Calibrate the microtome (clearance angle should be at 5, tissue thickness at 4 μm).
5.	Insert the paraffin wax block, lower the knife guard, unlock the wheel, coarse trim all blocks (15-30 μm) and place trimmed blocks back on the ice to cool.
6.	With one paraffine wax block, start to cut sections at a thickness of 4 μm, after obtaining a good ribbon, lock wheel, and cover blade with knife guard.
7.	Pick up tissue ribbon using tweezers and gently place it onto the water bath surface to remove wrinkles and then stand the clean slide in an upright position to allow the water to drain from the slide.
8.	Remove unused sections and clear the water surface regularly.
9.	Label slides.
10.	Place slides into the staining basket. Place basket into drying oven and set at 60 °C for one hour.

In round one of the modified Delphi methods, only one step fell below the mean of 3.75, at a value of 3.63, with high variability (SD of 1.69; Table 2). Eight experts responded to a survey in which they were asked to indicate whether a step is critical or not, rate the importance of each step using a 1-5 Likert Scale, and provide open feedback on each item on the list [4]. The purpose of this was to validate the steps of the procedure with multiple experts. This is important to ensure there is no bias from a single expert. A thematic analysis was conducted from the open feedback that was provided for each of the steps in the procedure and synthesized to create a general theme for each step. These themes were then used to alter any steps that fell below the threshold.

**Table 2:** Ratings from the modified Delphi method in rounds 1 and 2 The average rating for each step of the microtomy procedure (scale 1-5) is expressed as mean ± SD.

Steps	Round 1 (scale 1-5) # of participants: 8	Round 2 (scale 1-5) # of participants: 6
1.	3.63±1.69	3.83±1.69
2.	4.25±1.17	4.25±1.17
3.	4.75±0.71	4.75±0.71
4.	4.00±0.93	4.00±0.93
5.	4.88±0.35	4.88±0.35
6.	4.75±0.71	4.75±0.71
7.	4.50±0.54	4.50±0.54
8.	5.00±0.00	5.00±0.00
9.	5.00±0.00	5.00±0.00
10.	4.00±1.41	4.00±1.41

When examining the open feedback from round one, there was heterogeneity in the interpretation and, therefore, perceptions of the criticality of step one were due to the language used by stating that there was a need for ice, instead of stating that any cold surface would work. Revisions were made to step one of the microtomy procedure. The revised step was changed from "placing the tissue face down on ice" to "placing the tissue on a cold surface prior to cutting." In addition, the analysis of the written feedback from round one highlighted additional changes. More specifically, the participants' comments regarding steps three and eight were recorded. For step three, which states to lock the microtome and adhere to microtome safety, the feedback that was consistently provided was to ensure the microtome is locked before inserting the blade, as it is a high safety risk, and doing so will prevent any serious accidents. Step three now reads, "ensure the microtome is locked and adheres to microtome safety; place the blade in the blade holder and cover it with the knife guard to prevent serious accidents." For step eight, which asks to remove unused sections and clean the water surface regularly, the feedback was that cleaning the water and removing unused sections regularly prevents cross-contamination between patients and misdiagnosis. Step eight now reads, "remove unused sections and clear the water surface regularly to prevent cross-contamination." The language for all three steps was altered based on the feedback, and the new steps were submitted to the participants for review in round two. 

Six experts completed round two of the survey. The new rating for step one was 3.83, exceeding the 3.75 value, and was accepted (Table 2). The consensus was built on two rounds, eliminating the need for round three. The consensus is that all ten steps of the procedure will be kept and are indeed critical. The revised and finalized list of the ten steps is shown in Table 3.

**Table 3:** Finalized list of the 10 steps after round 2 of the modified Delphi method

Step	Description
1.	Place the tissue on a cold surface prior to cutting (cold wax make the tissue easier to cut).
2.	Fill the water bath and warm to approximately 46 °C.
3.	Ensure the microtome is locked and adhere to microtome safety. Place the blade in the blade holder and cover with the knife guard to prevent serious accidents.
4.	Calibrate the microtome (clearance angle should be at 5, tissue thickness at 4 μm).
5.	Insert the paraffin wax block, lower the knife guard, unlock the wheel, and coarse trim all blocks (15-30 μm) and place trimmed blocks back on the ice to cool.
6.	With one paraffine wax block, start to cut sections at a thickness of 4μm. After obtaining a good ribbon, lock wheel and cover blade with knife guard.
7.	Pick up tissue ribbon using tweezers and gently place it onto the water bath surface to remove wrinkles and then stand the clean slide in an upright position to allow the water to drain from the slide.
8.	Remove unused sections and clear water surface regularly to prevent cross-contamination.
9.	Label slides.
10.	Place slides into the staining basket. Place basket into drying oven and set at 60 °C for one hour.

Again, the open feedback from round two for step one was synthesized and used to refine and adjust step one for round two of the study. After round two, the consensus was reached, and the feedback from step one in round two was analyzed to generate the main theme of the feedback.

The main theme of the feedback that was received in round two after sending the revised version of the steps was that a cold surface will support the tissue structure, which will ultimately produce better overall sections. The main difference between the feedback provided in rounds one and two was that the participants did not think that placing the tissue on ice was necessarily required, but rather any cold surface would be sufficient.

## Discussion

Upon completing the think-aloud protocol, followed by a modified Delphi study, experts concluded that all the steps of the procedure are indeed critical. The experts' consensus validating the steps of the microtomy procedure is a critical first step in the development of any simulation exercise [4]. Current literature does not provide a universal standard for the microtomy procedure [1,2], and this was exhibited among the participants based on the responses provided in the open feedback section of the survey [6]. Although after two rounds, all experts were unanimous in their decision on whether the ten steps were indeed critical, the feedback demonstrated variability among the steps in terms of preference of tools, times, and temperatures. Therefore, this initial study provides two unique contributions to the field of simulation-based education in MLT training. First, to the best of our knowledge, this is the first study showing expert-based consensus on the steps necessary to perform microtomy. Second, this is the first study in the realm of MLT training and education to use a combination of the think-aloud protocol and Delphi methodology to generate educational content to build subsequent simulation exercises. 

Traditional education methods are limited due to patient safety, work-hour restrictions, instructor availability, and the cost of operating room time [7]. In the healthcare field, patient safety and complications are always a concern. Simulation-based education is an educational practice that introduces learners to an extensive learning environment, allowing them to develop knowledge and receive feedback without the pressure and cost of errors. It is defined as "the imitation or representation of one act or system by another" [8]. When practicing in a simulated setting, the trainees can develop the skills required at their own pace with unlimited repetition of specific scenarios catered to the skill [7]. However, before a simulation can be adequately and accurately developed, the content of the simulation must be accurate and validated. The result of this study is the first step to developing a virtual simulation training module to address the need to develop procedural knowledge (knowing how) related to protocols and the safe handling of equipment used during microtomy. The Delphi methodology was used by obtaining experts from MLTs to validate the steps of the microtomy procedure that was produced from the think-aloud method. The Delphi method has been used in numerous studies for simulation curriculum development [4].

A couple of limitations to this study should be noted. First, the experts that were used were limited to a single Canadian society, CSMLS. This may mean the findings are based on a single expert group rather than experts from various medical laboratories. Thus, the results may not have accounted for MLTs who may have conducted the procedure differently in different medical laboratory societies.

Second, the modified Delphi process was conducted via email in an asynchronous manner, which may have limited the opportunity to resolve any miscommunications among the experts. For instance, the main theme generated from the open feedback was in step one regarding the differences between whether placing the tissue face down on ice is critical or not, or if any cold surface is sufficient. Step one was revised after the feedback that was provided from round one, and although some experts may still have found it not critical, it did pass the 3.75 value, deeming it as a critical step. In addition, only six out of the eight initial participants from round one chose to complete the survey for the revised steps in round two. Due to this, there were fewer participants in round two than in round one.

Future research should focus on implementing a universal procedure for medical laboratory science procedures in not only microtomy but others as well, such as histological staining. This will prevent MLT training from being inconsistent, which leads to differences in producing a tissue sample for microscopic examination. Ultimately, this process could lead to a medical diagnosis, and it is critical for the tissue sample to be prepared consistently across various healthcare laboratories. Further efforts regarding this study will focus on the development of the virtual simulation module, which will be held on a learning management system known as Gamified Educational Network [9]. This type of simulation-based training may be advantageous because trainees can practice independently in a self-paced manner, which ultimately maximizes their clinical experience.

## Conclusions

The objective of this study was to validate the steps of the microtomy procedure in order to develop a virtual simulation module to teach microtomy. This study provides researchers and educators with a consistent number of steps for the microtomy procedure. Although simulation-based learning has been introduced into medical laboratory sciences, the methods used to develop simulation in this context are not well developed yet. This study has generated a stepwise list of the microtomy procedure. Therefore, this study offers two unique contributions to the growing field of simulation-based education in medical laboratory sciences training. First, it provides an expert-based consensus on the steps that are necessary to successfully complete a microtome procedure. Second, it introduces a tested methodology that can be employed to build a similar consensus for other skills and procedures to be simulated.
